# Significance of Glioma Stem-Like Cells in the Tumor Periphery That Express High Levels of CD44 in Tumor Invasion, Early Progression, and Poor Prognosis in Glioblastoma

**DOI:** 10.1155/2018/5387041

**Published:** 2018-08-23

**Authors:** Masahiro Nishikawa, Akihiro Inoue, Takanori Ohnishi, Shohei Kohno, Shiro Ohue, Shirabe Matsumoto, Satoshi Suehiro, Daisuke Yamashita, Saya Ozaki, Hideaki Watanabe, Hajime Yano, Hisaaki Takahashi, Riko Kitazawa, Junya Tanaka, Takeharu Kunieda

**Affiliations:** ^1^Department of Neurosurgery, Ehime University Graduate School of Medicine, Toon, Ehime 791-0295, Japan; ^2^Department of Neurosurgery, Washoukai Sadamoto Hospital, Matsuyama, Ehime 790-0052, Japan; ^3^Department of Neurosurgery, Ehime Prefectural Central Hospital, Matsuyama, Ehime 790-0024, Japan; ^4^Department of Molecular and Cellular Physiology, Ehime University Graduate School of Medicine, Toon, Ehime 791-0295, Japan; ^5^Division of Pathophysiology, Faculty of Pharmaceutical Sciences, Hokuriku University, Kanazawa, Ishikawa 920-1180, Japan; ^6^Division of Diagnostic Pathology, Ehime University Hospital, Toon, Ehime 791-0295, Japan

## Abstract

Glioblastoma multiforme (GBM) is the most aggressive malignant brain tumor and a subpopulation of glioma stem-like cells (GSCs) is likely responsible for the invariable recurrence following maximum resection and chemoradiotherapy. As most GSCs that are located in the perivascular and perinecrotic niches should be removed during tumor resection, it is very important to know where surviving GSCs are localized. Here, we investigated the existence and functions of GSCs in the tumor periphery, which is considered to constitute the invasion niche for GSCs in GBM, by analyzing expression of stem cell markers and stem cell-related molecules and measuring particular activities of cultured GSCs. In addition, the relationship between GSCs expressing particular stem cell markers and pathological features on MRI and prognosis in GBM patients was analyzed. We showed that GSCs that express high levels of CD44 are present in the tumor periphery. We also found that vascular endothelial growth factor (VEGF) is characteristically expressed at a high level in the tumor periphery. Cultured GSCs obtained from the tumor periphery were highly invasive and have enhanced migration phenotype, both of which were markedly inhibited by CD44 knockdown. Higher expression of CD44 in the tumor periphery than in the core was correlated with a highly invasive feature on MRI and was associated with early tumor progression and worse survival, whereas lower expression of CD44 in the tumor periphery corresponded to low invasion and was associated with longer survival. The low invasion type on MRI tended to show high levels of VEGF expression in the tumor periphery, thus presenting the tumor with high proliferative activity. These results imply the significance of GSCs with high levels of CD44 expression in the tumor periphery compared to the core, not only in tumor invasion but also rapid tumor progression and short survival in patients with GBM.

## 1. Introduction

Glioblastoma multiforme (GBM) is the most malignant type of primary brain tumor with a median survival of 15 months, even after maximum resection of the tumor followed by the current standard chemoradiotherapy [1]. The poor prognosis of this tumor type is considered to be largely due to glioma stem-like cells (GSCs), which constitute a small subpopulation of tumor cells in GBM and are responsible for early tumor progression, resistance to chemoradiotherapy, and aggressive invasion [2, 3]. After the initial treatment, most GBM tumors recur locally, arising from the periphery of the tumor cavity after resection [4, 5]. Recently, Munthe et al. reported that glioma cells in the tumor periphery of glioma patients have a stem cell phenotype [6], but the number of GBM patients in the study is very few and the role and pathophysiological functions of GSCs in the invasion zone of the tumor periphery have not been intensively investigated in GBM.

GSCs are located in a specific microenvironment called the “niche” where the stemness of the GSCs is maintained. Tumor initiation, survival, and invasion are dynamically regulated by intricate interactions between GSCs and various components of the microenvironment including host stromal cells [7, 8]. In GBM, two distinct regions, the perivascular niche and the hypoxic/perinecrotic niche, are considered specific niches where GSCs are enriched and their maintenance and survival are supported [9, 10]. GSCs located in the perivascular niche generally reside close to the endothelial cells of capillaries of the tumor neovasculature and play a central role in angiogenesis of highly disorganized vasculature to respond to a rapidly growing tumor [11, 12]. The hypoxic/perinecrotic niche, which is the hypercellular region around the necrosis, termed pseudopalisades, plays a crucial role in maintaining GSCs and promoting self-renewal of CD133-positive GSCs, thus expanding the GSC population in the entire tumor [13]. Although GSCs in these niches are thought to be critical for tumor proliferation, they are unlikely to be the main cause of tumor recurrence, particularly when the tumor is completely resected including the area of these niches.

On the other hand, the tumor invasive area at the outer edge of the tumor presents a specific microenvironment that may constitute another niche for GSCs [14, 15]. Recent genetic and molecular studies on GSCs have been performed by utilizing samples from the tumor core. Thus, little is known about the molecular features of GSCs located in the invasion niche in the tumor periphery.

In the present study, we examined the expression of stem cell markers and related molecules in tumor tissues obtained from two different sites in GBM, the core and the periphery of the tumor. By analyzing expression patterns of stem cell markers and stem cell-related molecules in the periphery compared to the core, we identified a stem cell marker and a stem cell-related molecule that were characteristically expressed at high level in the tumor periphery compared with the core. To clarify a functional role of the GSCs expressing such a specific stem cell marker at the site of the tumor periphery in tumor progression of GBM, we investigated the pathological functions of the cultured GSCs that were established by the primary cell culture of the tumor tissues obtained from the tumor periphery. In addition, to demonstrate a significant role of the GSCs expressing the specific stem cell marker in clinical aspects, we analyzed the relationship between the degree of expression of the specific marker and stem cell-related molecule in the periphery and the pathological feature on magnetic resonance imaging (MRI) and survival of GBM patients.

## 2. Materials and Methods

### 2.1. Patients and Study Design

Among 36 newly diagnosed GBM patients who underwent surgery according to the same treatment protocol at Ehime University Hospital between January 2014 and December 2016, we identified 13 patients with separate tumor samples obtained from two different sites, the core and the periphery of the tumor, with navigation-guided microsurgical techniques as described below. For the purpose of exploring stem cell markers and other molecules that were highly expressed in the tumor periphery, we first carefully analyzed the expression patterns of eight stem cell markers for GSCs (Nestin, CD133, CD15, CD44, Bmi-1, Sox2, Oct3/4, and CXCR4) and four stem cell-related molecules (HIF-1*α*, HIF-2*α*, VEGF, and transforming growth factor beta (TGF-*β*)) in both the core and periphery of the tumor in a small number of GBM patients (patient numbers 1 to 4 in Table 1). Next, focusing on the specific stem cell marker and stem cell-related molecule that we identified in the initial study, we investigated the pathological function of GSCs expressing the specific stem cell marker by using cultured GSCs that were established from the tumor tissue in the tumor periphery and analyzed clinical significance of GSCs expressing the specific marker in the pathological features on MRI and prognosis of patients with GBM.

### 2.2. Treatment and Tissue Sampling

All patients underwent craniotomy for tumor resection, followed by radiotherapy (60 Gy) and chemotherapy with temozolomide in accordance with the Stupp protocol [16]. Tumor resection was performed by multimodal navigation-guided microsurgery using fence-post catheter techniques [17]. Using the surgical techniques, tumor samples were separately obtained from the core and periphery of the tumor.

**Figure 1:**
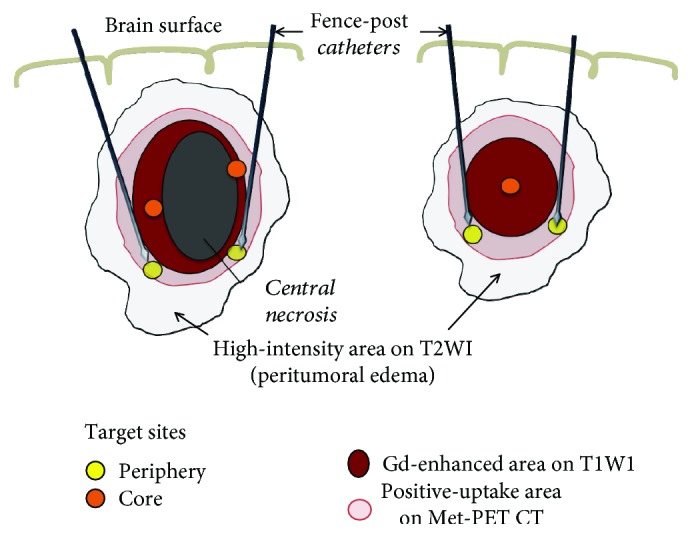
Navigation-guided surgical method for taking tumor samples from two sites, the periphery and core in GBM. The position of the tip of the fence-post catheters that are placed along the tumor border under echo-linked image-guided navigation is determined as a target for taking samples in the tumor periphery. The target area corresponds to just outside of the Gd-enhanced area and within the positive-uptake area on Met-PET. When the tumor showed a central necrosis, tumor tissues located outside the necrotic area were collected for the samples at the tumor core (left). Otherwise, the tumor samples at the core were obtained from the center of the resected tumor.

As a target for obtaining samples at the tumor periphery, we decided the position of the tip of the fence-post catheters that were placed along the tumor border using echo-linked image-guided navigation just before starting the tumor resection (Figure 1). The tumor samples at the core were obtained from the resected tumor mass. When the tumor had a large area of central necrosis (CN), tumors located outside the necrotic area were collected (Figure 1 left). The tumor samples were frozen and preserved at −80°C until use.

### 2.3. RNA Isolation and Quantitative Real-Time RT-PCR (qRT-PCR)

Total RNA was extracted from the tissue of each tumor sample using ISOGEN (Nippon Gene, Tokyo, Japan) according to the manufacturer's instructions. cDNA was synthesized from DNase I-treated RNA (1.5 *μ*g) by reverse transcription using M-MLV Reverse Transcriptase (Invitrogen, Tokyo, Japan) and oligo (dT) 15 primer. cDNA was diluted 1 : 3, and triplicate volumes of 1 *μ*l were used for qRT-PCR on a MJ Mini instrument (Bio-Rad, Hercules, CA) using FastStart Universal SYBR Green Master (Roche Diagnostic Japan, Tokyo, Japan). All gene-specific mRNA expression values were presented as relative expression levels normalized to the housekeeping (reference) gene glyceraldehyde 3-phosphate dehydrogenase (GAPDH). Quantification of gene expression was performed using ΔCt values, wherein ΔCt is defined as the difference between the target and reference gene Ct values. Validity of GAPDH as an internal control was certified by additional qRT-PCR using other housekeeping genes, beta-actin (ACTB), hydroxymethylbilane synthase (HMBS), and TATA-box binding protein (TBP).

The primer sequences are listed in Suppl.  Table S1.

### 2.4. Immunohistochemistry

Each tumor sample was fixed in formalin and embedded in paraffin. The blocks were sliced into 5 *μ*m-thick sections, which were deparaffinized in Histo-Clear (Cosmo Bio), hydrated in a graded series of alcohol, and subjected to heat-activated antigen retrieval. After blocking endogenous peroxidase activity, the tissue was incubated with CD44 monoclonal antibody (1 : 250, Cell Signaling Technology, number 3570S) for 4 hours at room temperature. Subsequently, the sections were washed and incubated with biotinylated secondary antibody for 30 minutes at room temperature. The reaction complexes were stained with diaminobenzidine and counterstained with hematoxylin.

For double-labeling immunofluorescence, sections were incubated with a mixture of two primary antibodies to CD44 (1 : 250, Cell Signaling Technology, number 3570S) and Nestin (1 : 250, EMD Millipore, ABD69) diluted in Tris-buffered saline containing 0.02% Tween 20 (TBST) and 0.1% bovine serum albumin in a humidified chamber overnight at 4°C. After washing with TBST, sections were treated with Cy3-conjugated anti-rabbit and DyLight 488-labeled anti-mouse IgG secondary antibodies (1 : 1000; Jackson ImmunoResearch, West Grove, PA). Hoechst 33342 (Sigma-Aldrich) was used for nuclear staining. The immunostained specimens were observed with a conventional microscope (BX52; Olympus, Tokyo, Japan).

### 2.5. Primary Cell Culture of Tumor Tissues Obtained from the Tumor Periphery

Tumor tissues were surgically obtained from the site of the tumor periphery of four GBM patients and were mechanically minced, digested with 0.1% trypsin, and then triturated with a Pasteur pipette. Cells were passed through a 70 *μ*m strainer (Falcon; Becton Dickinson Biosciences) and resuspended in high-glucose Dulbecco's modified Eagle's medium (DMEM) (Wako, Osaka, Japan) supplemented with 10% fetal bovine serum (FBS) and penicillin/streptomycin/amphotericin B mixture (Wako). Ability of the tumor cells in the primary cell culture to generate a sphere formation was examined by placing the cells in serum-free DMEM/Ham's F-12 (Wako) containing 10 *μ*g/ml insulin (Wako), 10 nmol/l recombinant human basic fibroblast growth factor, 10 nmol/l recombinant human epidermal growth factor, 5 *μ*mol/l heparin, N2 supplement (Wako), GlutaMAX supplement (GIBCO), and the penicillin/streptomycin/amphotericin B mixture (neural stem cell medium). Growth factors were purchased from PeproTech, London, UK. Primary tumor cells that were cultured in DMEM with 10% FBS, from which the sphere-forming cells (SFCs) were obtained by replacing the medium, were named as parent cells (PCs) for each SFCs.

### 2.6. Characterization of Sphere-Forming Cells (SFCs) as Tumor Stem Cells

To identify SFCs as cancer stem-like cells, mRNA expression of stem cell markers, Nestin, CD133, and CD44 on the SFCs was analyzed. Double-labeling immunofluorescence by using two antibodies to CD44 and Nestin was also performed as previously described. To investigate whether the SFCs had a potential for multilineage differentiation into neurons and astrocytes, the SFCs were cultured in the culture medium containing 10% FBS for 48 hours and then the immunohistochemistry using monoclonal antibodies TUJ1 (1 : 250, R&D Systems, MAB1195) and GFAP (1 : 250, Cosmo Bio, SML-ROI003) was performed. In addition, to evaluate in vivo tumorigenic activity of the SFCs, 1 × 10^4^ of the SFCs were transplanted into the brains of NOD/SCID mice. Generation of tumors in the mice brains was confirmed by MRI and then their brains were removed, sliced into 5 *μ*m-thick coronal sections, and the sections were stained with hematoxylin and eosin.

### 2.7. Cell Invasion and Migration Assays

Invasive activity of the cultured SFCs and PCs was assessed by the in vitro assay method using Falcon cell culture inserts (Becton Dickinson Biosciences, USA) and a reconstituted basement membrane, Matrigel (Becton Dickinson Biosciences), as previously described [18, 19]. Briefly, SFCs or PCs were suspended in DMEM containing 0.1% BSA and seeded onto the insert filters at a density of 5 × 10^4^ cells/insert. The insert was placed in the lower well of the Falcon 24-well plates containing 500 *μ*l of DMEM containing 1% FBS and incubated for 24 hours at 37°C under standard conditions.

Migration of the SFCs or PCs were assayed by the modified Boyden chamber method with 48-well microchemotaxis chambers (Nucleopore, Pleasanton, CA), as previously described [20, 21]. SFCs or PCs in DMEM containing 0.1% BSA (at a density of 1 × 10^4^ cells/ml) were placed in the upper well and DMEM containing 1% FBS was placed in the lower well. The filter was a polyvinylpyrolidone-free polycarbonate membrane with 8 *μ*m pores (EMD Millipore, Bedford, MA). The chamber was incubated for 6 hours at 37°C under standard conditions. In both assays, cells on the upper membrane surface were mechanically removed. Cells that had invaded or migrated to the lower side of the membrane were fixed, stained with 0.1% crystal violet, and put under a microscope (×400) to count the amount of cells in five random fields.

### 2.8. Treatment of Cultured SFCs with Small Interfering RNA (siRNA)

To clarify the effect of CD44 on the invasion and migration of the SFCs, the silencing effects of the CD44 gene with CD44 siRNA or control siRNA on invasion and migration of the SFCs were examined. CD44 siRNA sequences are follows: sense 5′-CCUCUGCAAGGCUUUCAAUAGTT-3′ and antisense 5′-CUAUUGAAAGCCUUGCAGAGGTT-3′. As a control for CD44 siRNA, we used a corresponding random siRNA sequence (5′-GCGCGCUUUGUAGGAUUCG dTdT). SFCs were transfected with CD44 siRNA using Lipofectamine 3000 Reagent (Invitrogen, Grand Island, United States) according to the manufacturer's instructions. After 24 hours' incubation of SFCs transfected with CD44 siRNA, invasion and migration assays were performed.

### 2.9. Analysis of Pathological Features of the Tumor Periphery on MRI

We evaluated the degree of invasiveness of the tumor in the periphery based on the findings of MRI. To make certain the features on MRI, we referred to the findings of 11C-methionine (Met) positron emission tomography (PET) studies and/or intraoperative 5-aminolevulinic acid- (5-ALA-) derived protoporphyrin IX (PpIX) fluorescence analysis for detecting the infiltrated tumor.

“High invasion” on MRI was defined as a tumor image that presented the findings of more than two features of the following three features. These included tumors with (1) an irregular or unclear margin, (2) extensive or diffusely surrounded peritumoral edema, and (3) extension to the contralateral side through the commissural fibers. Tumors presenting only one feature were evaluated for invasiveness through the Met-PET study, demonstrating that the tumor showing a much larger Met-uptake area than the Gd-enhanced area was highly invasive [22, 23] or had a higher fluorescence intensity of PpIX of the brain in the resection cavity. Tumors with a demarcated margin and with focal or scant edema were defined as “low invasion” on MRI. The degree of invasion was presented as high or low (Table 1).

This study was approved by the institutional review board of the Ethics Committee for Clinical Research of Ehime University Hospital, and written informed consent was obtained from each patient and/or a family member before initiating the study.

The mouse experiments were performed according to protocols approved by the Animal Care and Use Committees of Ehime University. The study protocol was approved by the Animal Care and Use Committees of Ehime University (permit number: 1708013).

### 2.10. Statistical Analysis

Parametric data were expressed as means ± standard deviations and compared using the Student's *t*-test. Nonparametric data were expressed as median values (interquartile range) and compared using the Mann–Whitney *U* test. Receiver operating characteristic curve analysis was used to determine a cutoff value for predicting the highly invasive type on MRI based on the periphery/core (P/C) ratio of CD44 expression. Kaplan-Meier plots, in which survival was expressed as a function of the time after surgery, were generated to estimate progression-free survival (PFS) and overall survival (OS) for each group, and the log-rank test was performed to determine statistically significant differences between groups.

## 3. Results

### 3.1. Clinical Features

The median age of the 13 patients identified in the present study was 66 years (range 44–79 years), and they presented with a median Karnofsky performance status (KPS) score of 70 (range 60–90). All patients underwent image-guided microsurgical resection followed by chemotherapy and radiation therapy. All tumors had a histopathologically confirmed diagnosis of GBM according to the World Health Organization classification system, and all tumors had no mutation in *IDH1*. Expression of O(6)-methylguanine-DNA methyltransferase (MGMT) and the Ki-67 SI in the core of the tumors in the 13 patients are summarized in Suppl.  Table S2. The extent of resection (EOR) was evaluated by volumetric analysis on MRI before and after surgery as previously described [24]. Gross total resection (GTR: 100% resection of the tumor volume), subtotal resection (95 to 100% resection), and partial resection (<95% resection) were achieved in 10 (76.9%), 2 (15.4%), and 1 patient (7.7%), respectively. After the initial standard therapy was completed, nine patients developed progression of the tumor, of whom seven patients showed local recurrence and two developed cerebrospinal fluid dissemination. One patient died of a sudden onset of pulmonary embolism 7 days after surgery. Three patients are alive and in good health without tumor recurrence. The surviving time of these patients is presented as overall survival (OS) in Table 1.

### 3.2. Expression of Stem Cell Markers and Stem Cell-Related Molecules in Two Different Sites, the Periphery and Core of the Tumor, in Four Patients

**Figure 2:**
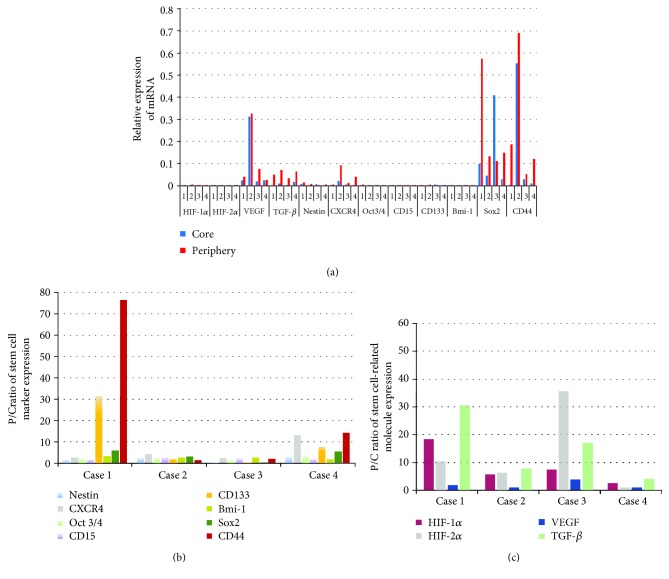
Expression of stem cell markers and stem cell-related molecules in the core and the periphery of the tumor in four glioblastoma (GBM) patients. (a) mRNA expression of eight stem cell markers (Nestin, CXCR4, Oct3/4, CD15, CD133, Bmi-1, Sox2, and CD44) and four stem cell-related molecules (HIF-1*α*, HIF-2*α*, VEGF, and TGF-*β*) in the core and the periphery was determined with qRT-PCR. The values are relative expression of mRNA of stem cell markers and molecules normalized to the GAPDH. (b) Expression of eight stem cell markers shown as the P/C expression ratio for each marker in each patient. The P/C ratios for the marker were calculated by dividing the amount of mRNA expression of the marker in the tumor periphery by that in the core for each patient. (c) Expression of four stem cell-related molecules shown as the P/C expression ratio for each molecule in each patient. The P/C ratios for the molecule were calculated as described for the stem cell markers. The numbers on the *x*-axis are the patient numbers.

We analyzed the mRNA expression of eight stem cell markers (CD133, CD15, CD44, Nestin, Bmi-1, Sox2, Oct-3/4, and CXCR4) and four stem cell-related molecules (HIF-1*α*, HIF-2*α*, VEGF, and TGF-*β*) in two different sites of tumors in four patients. Most stem cell markers were expressed at only a low level in the both core and periphery of the tumor, except for Sox2 and CD44, which showed high expression in both sites, and CXCR4, which showed slightly higher expression in the periphery (Figure 2(a)). Regarding the stem cell-related molecules, VEGF and TGF-*β* were expressed at a relatively high level, with slightly higher expression of VEGF in both the core and periphery than TGF-*β* (Figure 2(a)). To identify specific stem cell markers that may play an important role in the tumor periphery, we analyzed expression patterns of the stem cell markers by evaluating the intensity and patterns of the ratio of expression of these markers in the periphery to expression in the core (P/C ratio). Among eight stem cell markers, CD44 showed the highest P/C ratio in two patients, in marked contrast to two other patients who had a low P/C ratio (Figure 2(b)). Although CD133 also showed a high P/C ratio in the same patients as those with a high CD44 P/C ratio, the expression of CD133 mRNA was too low in all patients. Thus, we focused on CD44 as a stem cell marker for further analysis.

The P/C ratios for stem cell-related molecules other than VEGF showed various values among patients (Figure 2(c)). TGF-*β* showed high P/C ratios in two patients (numbers 1 and 3), but immunohistochemical analysis showed that the expression was partly derived from tumor-associated macrophages (data not shown). Unlike stem cell markers expressed on glioma stem cells, it is considered that these molecules closely participate in not only regulating the biological function of the stem cells but also modifying the condition of the microenvironment. So, we put much focus on the molecule that was expressed in the highest quantity. As a result, we selected VEGF for further investigation in the present study.

### 3.3. Immunohistochemical Study

**Figure 3:**
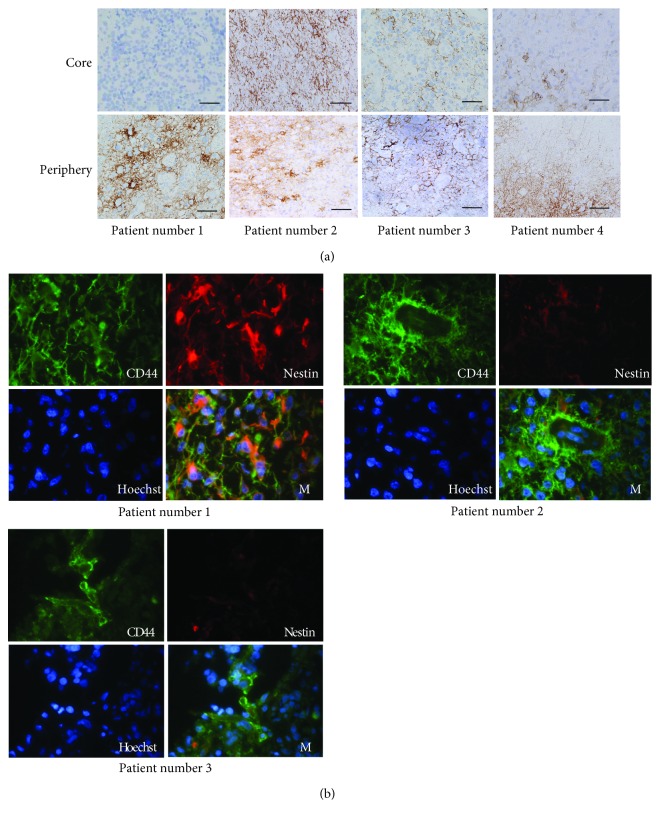
Immunohistochemical expression of the stem cell markers CD44 and Nestin. (a) Immunostaining for CD44 in the core and the periphery of the tumor in four GBM patients. The number of tumor cells showing positive staining for CD44 in the periphery was much higher than that in the core in tumors with a high P/C ratio for CD44 expression (patient numbers 1 and 4) (scale bar: 100 *μ*m) (×400). (b) Double-labeling immunofluorescence. CD44 (green) and Nestin (red) were colocalized (M: merge) at the cell membrane in some GBM cells in the tumor periphery. Nuclei were labeled with Hoechst (blue).

To confirm the expression of CD44 at the cellular level, immunohistochemical expression of CD44 in the core and the periphery of the tumors was examined. CD44 showed positive immunostaining that was much stronger in the tumor cells in the periphery than in the core in patients with much higher CD44 mRNA expression in the periphery than in the core (patients with a high P/C ratio). However, the difference in the degree of positive staining for CD44 between the periphery and the core was not remarkable in the patients with slightly higher CD44 mRNA expression in the periphery compared to the core (patients with a low P/C ratio) (Figure 3(a)). Double-labeling immunofluorescence disclosed that about a half of the CD44-positive cells in the periphery in patients with a high P/C ratio also expressed Nestin, but coexpression of CD44 and Nestin was not observed in most tumor cells in the periphery in the patients with a low P/C ratio (Figure 3(b)).

### 3.4. Identification of SFCs as Glioma Stem-Like Cells

**Figure 4:**
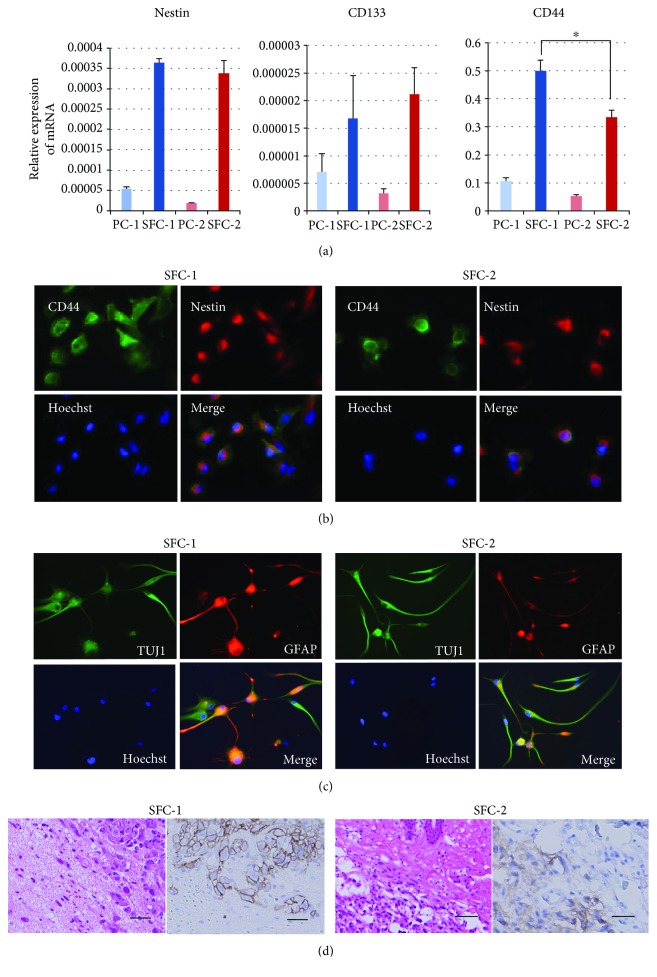
Characterization of sphere-forming cells (SFCs) as glioma stem-like cells. (a) Both SFC-1 and SFC-2 expressed much more CD133 and Nestin mRNA than those parent cells (PC-1 and PC-2). SFC-1 expressed CD44 at a significantly higher level than SFC-2. ^∗^ *p* < 0.01. (b) CD44 (green) and Nestin (red) were colocalized at the cell membrane in both SFC-1 and SFC-2. (c) Both SFC-1 and SFC-2 showed the capacity for multilineage differentiation into neurons (TUJ1; green) and astrocytes (GFAP; red). The nuclei are labeled with Hoechst (blue). (d) Both SFCs transplanted in the mouse brain generated invasive tumors (HE × 400), in which tumor cells in the periphery were positively stained for CD44 (bar 100 *μ*m) (×400).

In the primary cell culture of the tumor tissues obtained from the tumor periphery of four GBM patients, we obtained two SFCs (SFC-1 and SFC-2), both of which expressed much higher amounts of mRNA of neural stem cell markers CD133, Nestin, and CD44 than each PCs (Figure 4(a)). The expression level of CD44 in SFC-1 was higher than that in SFC-2. Double-labeling immunofluorescence demonstrated colocalization of CD44 and Nestin in both SFCs (Figure 4(b)). When these SFCs were cultured in the serum-containing medium, they were positively stained for TUJI and GFAP antibodies (Figure 4(c)). In addition, transplanted SFCs in the brains of NOD/SCID mice generated a highly invasive tumor whose invasion front was positively stained for CD44 (Figure 4(d)).

### 3.5. Invasive and Migratory Activities of Cultured SFCs and Inhibitory Effects of CD44 Knockdown on These Activities

**Figure 5:**
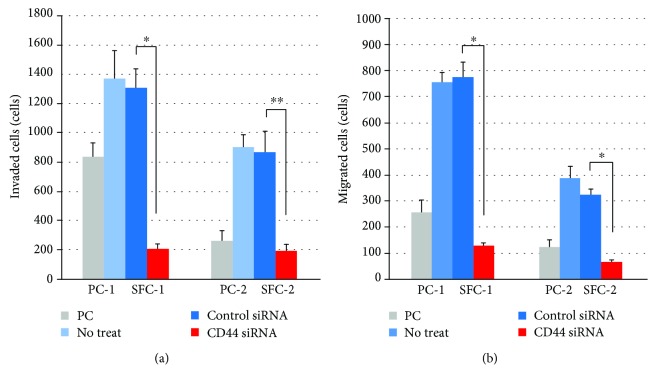
Invasive and migratory activities of cultured SFCs and PCs and inhibitory effect of CD44 knockdown with CD44 siRNA on their activities. In both invasion (a) and migration (b), cultured SFCs (SFC-1 and SFC-2) showed much higher activity than each PCs (PC-1 and PC-2). These high activities of invasion and migration of SFCs were significantly inhibited by knockdown of CD44 with the siRNA. ^∗^ *p* < 0.001 and ^∗∗^ *p* < 0.005.

In vitro invasion and migration studies disclosed that both SFC-1 and SFC-2 had much higher invasive and migratory activities than their PCs (PC-1 and PC-2) (Figures 5(a) and 5(b)). SFC-1 showed much stronger activity than SFC-2 in both invasion and migration. These high activities of invasion and migration of both SFC-1 and SFC-2 were significantly inhibited by CD44 knockdown with the siRNA (Figures 5(a) and 5(b)).

### 3.6. Expression of CD44 and VEGF in the Core and the Periphery of the Tumor in 13 GBM Patients

**Figure 6:**
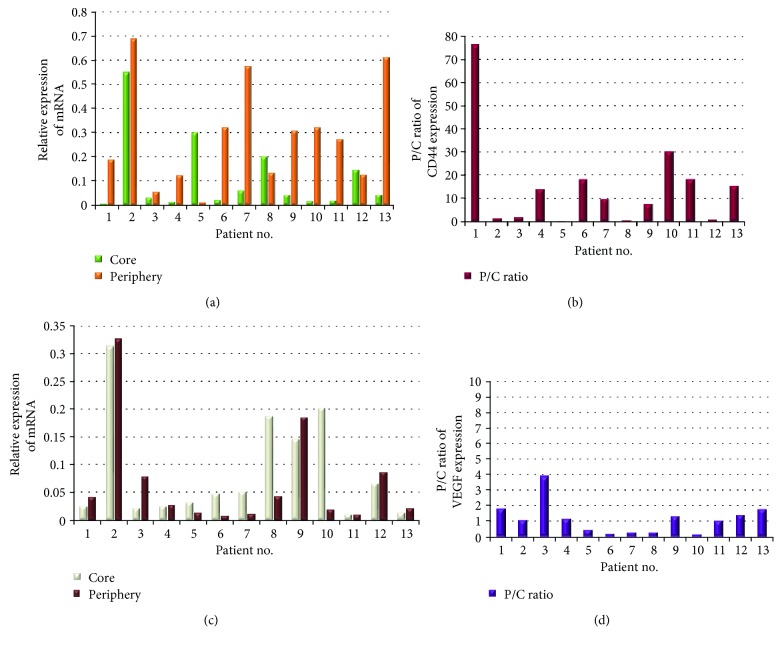
Expression of the stem cell marker, CD44, and the stem cell-related molecule, VEGF, in 13 GBM patients. (a) mRNA expression of CD44 in the tumor core and periphery was determined with qRT-PCR. The values are relative expression of mRNA normalized to the GAPDH. (b) Expression of CD44 represented by the P/C ratio. The P/C ratios for CD44 were calculated by dividing the amount of mRNA expression of CD44 in the tumor periphery by that in the core for each patient. (c) mRNA expression of VEGF in the tumor core and periphery. The expression and the level were determined as described for CD44. (d) Expression of VEGF represented by the P/C ratio. The P/C ratios for VEGF were determined as described for CD44.

We extensively investigated the expression of CD44 and VEGF in both the core and periphery of the tumor in 13 patients. Ten of 13 patients showed higher expression of CD44 in the periphery than in the core (Figure 6(a)). Among them, eight patients had very high P/C ratio values (>7.48; Figure 6(b)). On the other hand, three patients presented with higher expression of CD44 in the core than in the periphery.

Six patients showed higher expression of VEGF in the periphery than in the core (Figure 6(c)). However, because the difference in the amount of VEGF expression between the periphery and the core was low, no significant difference in the P/C ratio among the patients was found (Figure 6(d)).

### 3.7. Evaluation of Invasive Feature on MRI in the Periphery of GBM

**Figure 7:**
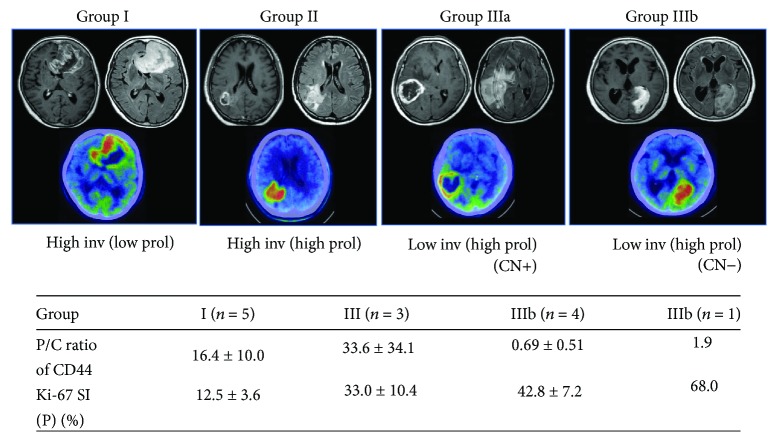
Representative combined images of MRI and PET-computed tomography for four groups of features with different intensities representing invasion and proliferation in the tumor periphery. Inv: invasion, prol: proliferation, CN: central necrosis. In all images, the upper left is T1-Gd, the upper right is a fluid-attenuated inversion recovery image, and the lower image is Met-PET. Values at the bottom are the mean ± SD of the P/C ratio for CD44 expression (upper row) and the Ki-67 SI in the tumor periphery (lower row). *n*: number of patients.

We evaluated the degree of invasiveness of the tumor in the periphery by analyzing characteristic features on MRI in 13 patients. Five patients who showed almost the same findings on MRI of an irregular tumor margin with necrosis and diffuse peritumoral edema (Group I) were judged as the high invasion (Hi-I) type. All Hi-I type tumors showed low Ki-67 SI (12.5 ± 3.6%) in the tumor periphery. Three patients with extensive, diffuse edema and various configurations of a Gd-enhanced tumor (Group II) were considered the Hi-I type with high proliferative activity (Ki-67 SI (33 ± 10.4%)) in the tumor periphery. Four patients whose tumor showed large central necrosis (CN) with a ring-like enhanced wall with a relatively smooth margin and was accompanied by only focal edema on MRI (Group IIIa) were considered the low invasion (Lo-I) type, and they all presented with high proliferative activity (Ki-67 SI (42.1 ± 7.2%)) in the tumor periphery. One patient with a solid tumor mass and a clear margin without CN and with slight, focal edema (Group IIIb) was also considered the Lo-I type but with much higher proliferation (Ki-67 SI (68%)). Representative MR and PET scan images of each group are shown in Figure 7. Table 1 shows the intensity of tumor invasion as seen on MRI, the proliferative activity shown by Ki-67 SI in the tumor periphery, CD44 and VEGF expression values presented as both mRNA in the periphery and the P/C ratio, and the clinical outcome including PFS and OS in 13 patients.

### 3.8. Relationship between CD44 and VEGF Expression in the Tumor Periphery and Pathological Features on MRI and the Clinical Outcome

We next analyzed the relationship between the degree of CD44 and VEGF expression in the tumor periphery and pathological features on MRI and clinical outcomes.

**Figure 8:**
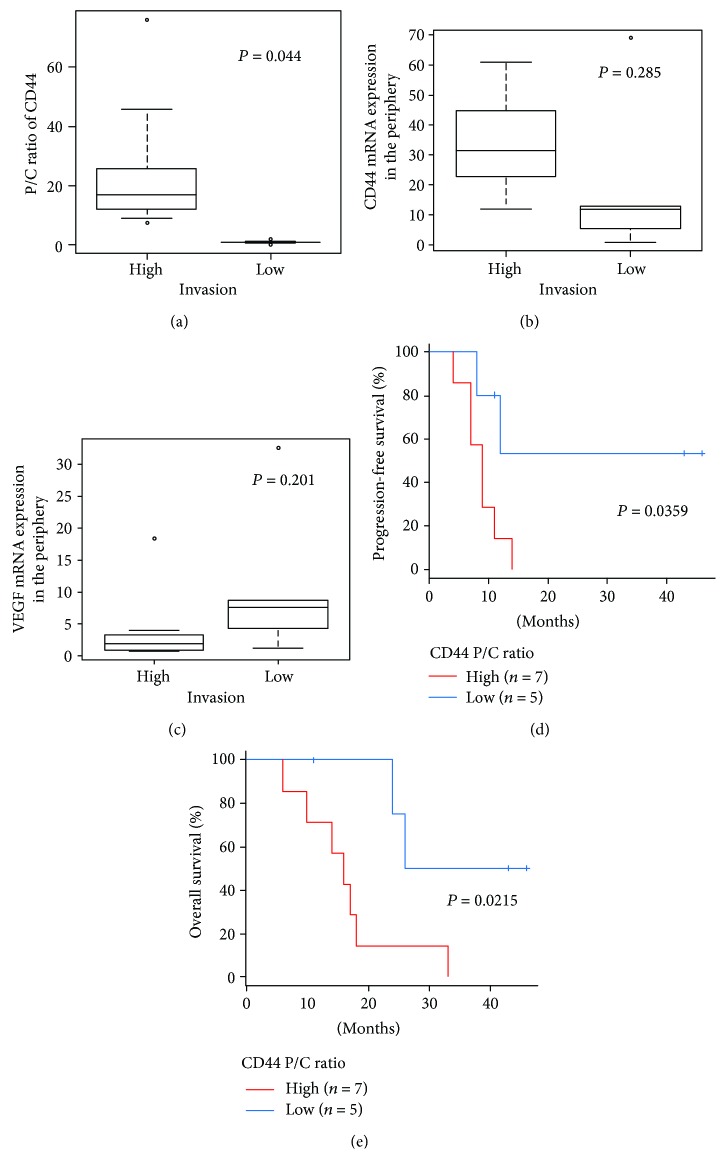
(a) Relationship between the P/C ratio for CD44 expression and tumor invasiveness as seen on MRI. Patients with a high P/C ratio showed a significant, more highly invasive type on MRI than those with a low P/C ratio (*p* = 0.0441, *t*-test). (b) Relationship between the level of CD44 mRNA expression in the tumor periphery and tumor invasiveness as seen on MRI. The level of CD44 mRNA expression in the tumor periphery did not correlate with invasiveness on MRI (*p* = 0.285, *t*-test). (c) Relationship between the level of VEGF mRNA expression in the tumor periphery and tumor invasiveness as seen on MRI. The low invasive type on MRI tended to have higher amounts of VEGF expression in the tumor periphery (*p* = 0.201, *t*-test). (d, e) Kaplan-Meier survival curves for the P/C ratio for CD44 expression predicting PFS (d) and OS (e). Patients with a high P/C ratio for CD44 expression showed significantly earlier tumor progression and worse survival than those with a low P/C ratio (*p* = 0.0359 and 0.0215, respectively, log-rank test).

As presented in Table 1, all eight patients with a high P/C ratio for CD44 expression showed the Hi-I type on MRI (Groups I and II), whereas all five patients with a low P/C ratio for CD44 showed the Lo-I type (Groups IIIa and IIIb). The difference in the P/C ratio for CD44 expression between Hi-I and Lo-I types on MRI was statistically significant (Hi-I (24.0 ± 22.31) versus Lo-I (0.93 ± 0.7), *p* = 0.0441) (Figure 8(a)). On the other hand, we found no significant difference in the level of CD44 mRNA expression in the tumor periphery between the two types (Hi-I (33.93 ± 17.22) versus Lo-I (20.03 ± 27.84), *p* = 0.285) (Figure 8(b)). The Lo-I type on MRI tended to show higher amounts of VEGF expression in the periphery (Lo-I (10.84 ± 12.5) versus Hi-I (3.92 ± 5.85), *p* = 0.201) (Figure 8(c)).

To evaluate the relationship between the degree of CD44 expression in the tumor periphery and prognostic outcome, Kaplan-Meier survival plots were generated for two groups of patients: those with a high P/C ratio for CD44 and those with a low P/C ratio for CD44. PFS and OS in each group were statistically analyzed (Figures 8(d) and 8(e)). Patients with a high P/C ratio for CD44 had significantly earlier tumor progression and a worse outcome than those with a low P/C ratio for CD44 (PFS: 9 ± 4.24 months (high P/C ratio) versus not available (NA) (low P/C ratio), *p* = 0.0359; OS: 16 ± 3.78 months (high P/C ratio) versus 26 ± 5.22 months (low P/C ratio), *p* = 0.0215). No significant differences were found in age, KPS score, immunostaining for MGMT, or Ki-67 SI (C) between the two groups with high and low P/C ratios for CD44 expression. Patients with high VEGF expression in the tumor periphery tended to have longer survival without tumor progression than those with low VEGF in the tumor periphery.

## 4. Discussion

In the present study, we demonstrated that GSCs with high expression of the stem cell marker, CD44, were present in the periphery of GBM. The periphery is a border area between the outer edge of the tumor and the normal brain, constituting the tumor-border (invasion) niche. Several stem cell markers, including eight markers examined in this study, have been identified as GSC markers, and the expression of these markers in tumor tissue and primary culture of GBM has been investigated. Bien-Möller et al. [25] analyzed the expression of seven stem cell markers as well as differentiation and microglia markers and demonstrated that CD44, ELF4, Nanog, and Nestin were elevated at both mRNA and protein levels in GBM, but only CD133 and Nestin were associated with survival rates. However, no marker has been established as a universal GSC marker [26], even CD133 still remains controversial [27, 28]. In addition, most of these studies were based on tumor samples obtained from the core of the GBM tumor.

Consequently, to explore the possible importance of stem cell markers with characteristically high expression in the tumor periphery, we analyzed the expression pattern of eight stem cell markers in both the core and periphery of the tumor in GBM patients. Expression of the eight stem cell markers was represented as the P/C ratio to illustrate expression in the tumor periphery compared to the core.

Most markers except CD44 and CD133 showed almost the same values, with a P/C ratio of about 2.0 in all patients. This result suggests that stem-like cells with the same function as GSCs in the tumor core also exist in the periphery. The present double immunostaining study showed that CD44 and Nestin were colocalized at the cell membrane of the tumor cells in the tumor periphery, demonstrating existence of stem cells expressing CD44 in the invasion area. Guadagno et al. [29] reported that coexpression of CD44 and Nestin in GBM is specifically observed at the cell membrane in undifferentiated stem-like cells. Our immunohistochemical study, showing that the tumors with a high P/C ratio for CD44 contained much more GSCs expressing CD44 in the tumor periphery compared with those with a low P/C ratio for CD44, may suggest that there are many more GSCs in the microenvironment with upregulating CD44 expression on GSCs than those in the microenvironment without stimulation for CD44 expression. This may be a reason we could not obtain sphere-forming cells by the primary cell culture of the tumor tissues expressing a low P/C ratio for CD44. To investigate the existence and biological functions of GSCs expressing CD44 in the tumor periphery of GBM, we tried to establish stem-like cells by placing the cells in primary culture from the tumor tissues in the tumor periphery in neural stem cell culture medium. Among the cell cultures from four tumor tissues, we obtained two SFCs (SFC-1 and SFC-2), both expressing stem cell markers CD133 and Nestin at almost the same levels. On the other hand, SFC-1 expressed much more highly CD44 than SFC-2. These SFCs had abilities to show multilineage differentiation to neurons and astrocytes and make a rapid tumorigenesis by the mouse xenograft model, thus demonstrating that these SFCs were identified as GSCs. In vitro invasion and migration studies disclosed that these cultured GSCs had high invasive and migratory activity, and GSC-1 expressing more CD44 had much stronger activities in both invasion and migration than GSC-2 expressing less CD44. In addition, the fact that their activities were significantly decreased by knockdown of the CD44 gene strongly indicates that CD44 has an essential activity of enhancing invasion and migration of the GSCs. These results indicate that GSCs with a higher expression of CD44 in the tumor periphery than the core are actually present in the tumor-border niche in GBM and they have high invasive and migratory activities that are promoted by CD44.

CD44 is a cell membrane glycoprotein that is involved in various cellular processes including cell motility, proliferation, apoptosis, and angiogenesis [30, 31]. CD44 is expressed in many cancers, including lung, breast, colon, prostate, and brain cancer [32–34]. In particular, CD44 expression in GBM enhances the invasion of GBM by promoting tumor cell migration through cell-extracellular matrix interactions [35]. Ariza et al. [36] described that GBM expressing CD44 showed high invasiveness, but meningioma lacking the CD44 expression could not infiltrate the brain extracellular matrix. Recent studies have described CD44 as a marker of GBM cancer stem cells, also known as glioma-initiating cells or GSCs, and CD44 expression is enriched in GSCs [37]. In addition to the invasive activity that is based on the interaction of the extracellular domain of CD44 with the extracellular matrix, CD44 can act as an intracellular signaling molecule by enhancing the expression of the CD44 intracellular domain to maintain and increase the stemness of these stem-like cells [38].

Little is known about the clinical significance of the GSCs highly expressing CD44 with such multifunctional activities, particularly, in the tumor progression of GBM. To clarify the role of the GSCs that highly expressed CD44 in the tumor progression, we investigated the possible association between the degree of expression of CD44 in the tumor periphery and imaging features on MRI and survival in GBM patients.

From our analysis of CD44 expression in 13 patients, we found that patients with a high P/C ratio for CD44 expression showed the Hi-I phenotype as seen on MRI and vice versa. These results indicate that the presence of GSCs that express higher levels of CD44 in the tumor-border zone than in the core may contribute to the invasive nature of the tumor as the GSCs highly expressing CD44 showed high invasive activity in in vitro studies. In particular, among Hi-I tumors, those of the high proliferation type in the periphery (Group II) showed much earlier progression. This group showed very high values in not only the P/C ratios for CD44 but also the relative amounts of CD44 mRNA; they also showed relatively low amounts of VEGF expression in the tumor periphery. The reason why some Hi-I type of GSCs expressing high levels of CD44 also show strong tumor proliferation is not clear. In our first study, the tumor with high invasion and high Ki-67 SI expressed CD133 at a distinctly high level (Figure 2(b)). As Brown et al. [39] reported, the worst type of GBM may result from alteration of the intratumor equilibrium of CD44+ and CD133+ subpopulations of GSCs, which may be influenced by environmental factors, resulting in a shift in the balance from CD44+ to CD133+ and hence, high proliferative activity.

A high P/C ratio for CD44 expression in GSCs was also strongly associated with not only early tumor progression but also worse survival. We found no significant difference in age, KPS score, MGMT immunostaining, or Ki-67 SI in the tumor core between the two groups with a high or low P/C ratio for CD44 expression. We also found no significant difference in OS between patients younger than 65 years and those who were older, patients with a KPS less than or equal to 70 and those with a KPS greater than or equal to 80, patients with an EOR of GTR and those with non-GTR (subtotal resection and partial resection), or patients with positive and those with negative MGMT staining. Consequently, a high P/C ratio for CD44 with a cutoff value of 7.48 for high invasiveness on imaging may also be a novel marker for poor prognosis.

CD44 is highly expressed in the mesenchymal subtype of GBM [40], and expression of CD44 has been indicated to be correlated with poor survival [41]. However, Pietras et al. reported that CD44 expression only in the perivascular niche of the proneural subtype of GBM is correlated with aggressive growth and poor prognosis [38]. On the other hand, gene coexpression analysis demonstrated that tumors with a CD133 coexpression module signature (CD133-CMS) are enriched for the proneural GBM subtype, which shows enhanced proliferation, and those with the CD44 coexpression module signature (CD44-CMS) are enriched for the mesenchymal subtype and show enhanced invasion. However, the study showed no long-term survival difference between CD44-CMS and CD133-CMS patients [42]. This result may be due to evaluation of the expression of markers using tissue samples obtained from the core of the tumor. In this context, Bhat et al. [43] demonstrated that NF-*κ*B-induced mesenchymal differentiation (transition) from the proneural subtype of GBM to the mesenchymal subtype was associated with increases of CD44 subpopulations and radio resistance, resulting in shorter survival in GBM patients. Taken together, it is strongly suggested that increased expression of CD44 may become a predictor for poor survival in GBM.

In addition to CD44, VEGF was the most highly expressed molecule, not only in the tumor periphery but also in the core. VEGF promotes angiogenesis, which is a key process of vascular formation by GSCs. In our study, although a high level of VEGF expression in the tumor periphery was not always associated with increased proliferative activity of the tumor in the periphery, we demonstrated that GBMs with the Lo-I phenotype as seen on MRI tended to show the high proliferative activity. These tumors also expressed high levels of VEGF in the periphery. The data in the present study also showed that Hi-I and high proliferative tumors expressed HIF-1*α* and CD133 most strongly, whereas Lo-I and high proliferative tumors expressed much more HIF-2*α* than HIF-1*α* (Figure 2(c)). These data suggest that the microenvironment around GSCs with the Hi-I type is much more hypoxic than the tumor with high proliferative activity. These phenomena may resemble the pathophysiology of the migration/proliferation dichotomy or the “Go or Grow” theory [44, 45].

The present study demonstrates the existence of GSCs in the tumor periphery of GBM, corresponding to the tumor-border (invasion) niche where the blood-brain barrier is not disrupted and thus, a much more hypoxic microenvironment is present compared with the peripheral contrast-enhancing layer of GBM [46]. In highly invasive GBM, GSCs located in such an invasion niche are thought to promote tumor invasion by increasing the expression of CD44, resulting in early tumor progression or dissemination. On the other hand, a high level of VEGF in the tumor periphery may suppress the invasive activity of CD44.

The relatively small number of patients, in whom the tumors are accurately taken from two different sites, is a critical limitation for particularly performing statistical analysis in the clinical study that should be mentioned. More extensive analysis with an increased number of patients would be required to obtain a definite conclusion and before application to clinical practice.

## 5. Conclusions

We demonstrated that GSCs highly expressing CD44 exist in the invasion niche at the tumor periphery of GBM. The cultured GSCs with a high expression of CD44 obtained from the tumor periphery had a high invasive activity that is enhanced by CD44. The higher expression of CD44 in the tumor periphery compared to the tumor core (high P/C ratio for CD44 expression) was correlated to early tumor progression and short survival of GBM patients. On the other hand, the stem cell-related molecule, VEGF, in the tumor periphery was highly expressed in the low invasive type of tumors, thus being considered to have a role in tumor proliferation in the tumor periphery. In the future, not only clarification of the predominant expression of CD44 in the tumor-border zone but also functional analysis of CD44 at the molecular level may enable researchers and clinicians to present the specific treatment strategy for each patient, more exactly predict the clinical prognosis, and develop a novel targeted therapy for GSCs with a high expression of CD44 to eradicate the tumor.

## Figures and Tables

**Table 1:** Summary of clinical features on MRI, Ki-67 SI (in the periphery), CD44, and VEGF expression values and clinical prognosis including survival and outcome in 13 patients with GBM.

Patient	Features on MRI			Ki-67 SI (P)		CD44		VEGF		Survival and outcome		
Number	Group	Intensity of invasion	Central necrosis	%	Proliferative intensity	mRNA in periphery	P/C ratio	mRNA in periphery	P/C ratio	PFS (months)	OS (months)	Status
1	II	H	−	26.1	H	0.19	76	0.041	1.75	7	10	D
2	IIIa	L	+	33.3	H	0.69	1.25	0.33	1	46	46	A
3	IIIb	L	−	68.3	H	0.053	1.9	0.076	4	43	43	A
4	I	H	+	16.8	L	0.12	14.1	0.025	1	14	17	D
5	IIIa	L	+	42	H	0.007	0.02	0.012	0.4	12	24	D
6	II	H	+	44.9	H	0.32	18.4	0.007	0.14	7	16	D
7	I	H	−	7.1	L	0.58	9.8	0.01	0.2	9	33	D
8	IIIa	L	+	42.3	H	0.13	0.65	0.043	0.23	8	26	D
9	I	H	+	13.7	L	0.31	7.48	0.18	1.3	NA	NA	D
10	I	H	+	11.6	L	0.32	32.8	0.018	0.1	9	18	D
11	I	H	−	13.2	L	0.27	18	0.009	0.8	11	14	D
12	IIIa	L	+	50.8	H	0.12	0.85	0.086	1.3	11	11	A
13	II	H	−	28	H	0.61	15.5	0.02	1.7	4	6	D

Ki-67 SI (P): Ki-67 staining index (in the tumor periphery), VEGF: vascular endothelial growth factor, P/C ratio: periphery/core ratio, PFS: progression-free survival, OS: overall survival, H: high, L: low, NA: not assessed, status: D (dead), A (alive).

## Data Availability

The data used to support the findings of this study are available from the corresponding author upon request.

## Supplementary Materials

Supplementary Materials Supplementary Table 1: oligonucleotide primers used in this study for qRT-PCR. Supplementary Table 2: summary of clinical features.
